# Applying queueing theory to evaluate wait-time-savings of triage algorithms

**DOI:** 10.1007/s11134-024-09927-w

**Published:** 2024-09-21

**Authors:** Yee Lam Elim Thompson, Gary M. Levine, Weijie Chen, Berkman Sahiner, Qin Li, Nicholas Petrick, Jana G. Delfino, Miguel A. Lago, Qian Cao, Frank W. Samuelson

**Affiliations:** The U.S. Food and Drug Administration, White Oak, MD USA

## Abstract

In the past decade, artificial intelligence (AI) algorithms have made promising impacts in many areas of healthcare. One application is AI-enabled prioritization software known as computer-aided triage and notification (CADt). This type of software as a medical device is intended to prioritize reviews of radiological images with time-sensitive findings, thus shortening the waiting time for patients with these findings. While many CADt devices have been deployed into clinical workflows and have been shown to improve patient treatment and clinical outcomes, quantitative methods to evaluate the wait-time-savings from their deployment are not yet available. In this paper, we apply queueing theory methods to evaluate the wait-time-savings of a CADt by calculating the average waiting time per patient image without and with a CADt device being deployed. We study two workflow models with one or multiple radiologists (servers) for a range of AI diagnostic performances, radiologist’s reading rates, and patient image (customer) arrival rates. To evaluate the time-saving performance of a CADt, we use the difference in the mean waiting time between the diseased patient images in the with-CADt scenario and that in the without-CADt scenario as our performance metric. As part of this effort, we have developed and also share a software tool to simulate the radiology workflow around medical image interpretation, to verify theoretical results, and to provide confidence intervals for the performance metric we defined. We show quantitatively that a CADt triage device is more effective in a busy, short-staffed reading setting, which is consistent with our clinical intuition and simulation results. Although this work is motivated by the need for evaluating CADt devices, the evaluation methodology presented in this paper can be applied to assess the time-saving performance of other types of algorithms that prioritize a subset of customers based on binary outputs.

## Introduction

Artificial intelligence (AI) and machine learning (ML) technologies have the potential to transform healthcare in many ways. One emerging area is AI/ML-enabled Software as a Medical Device (SaMD) in radiological imaging to triage patient images with time-sensitive findings for image interpretation [19]. These devices are known as computer-aided triage and notification (CADt) devices, by which medical images labeled as positive by an AI algorithm are given a higher priority in the radiologist’s reading queue than those labeled as AI-negative. The major benefit of a CADt device is to increase the likelihood of timely diagnosis and treatment of severe and time-critical diseases such as large vessel occlusion (LVO), intracranial hemorrhage (ICH), pneumothorax, etc. In 2018, the U.S. Food and Drug Administration (FDA) granted marketing authorization to the first CADt device for potential LVO stroke patients via the *de novo* pathway [17]. Since then, multiple studies have shown improvements in patient treatment and clinical outcomes due to the use of CADt devices [1, 7, 8, 20]. Most of these analyses focus on the diagnostic performance when evaluating these CADt devices, but a quantitative estimate of time savings for truly diseased patient images in a clinical environment remains lacking.

During the development of AI/ML algorithms and devices for clinical diagnosis, high emphasis is placed on the diagnostic performance (e.g., sensitivity, specificity, receiver operating characteristic (ROC) analysis, etc.) of the algorithm. Although the operational implications within a clinical environment receive less attention, a few studies exist in the literature investigate how a triage device can be deployed to maximize patients’ health outcome and clinicians’ workload. For example, using a queueing-based Markov decision process, Shi et al. investigated whether and how a clinic should adopt and integrate new diagnostic tests for certain subgroups of patients [13]. To decide whether a new test should be adopted, their framework defines a region in the ROC space in which the new test should achieve. The authors also provide a few alternative patient-routing policy to improve patient outcomes and reduce workload of the clinicians. For this work, however, we are interested in how much time is saved for the diseased patients due to a CADt device when operating at a fixed sensitivity and specificity threshold to all patient images in the reading list (not just a subset of images) and without any changes in the current workflow practice. Other studies integrate queueing theory into the design of a triage device. Sun et al. considered the cost of delay in a triage process where the time to inquire patient information for triage is non-negligible [16]. Their work considers a queueing model with two priority classes and one server, which is limited when it is applied to evaluate the use of a CADt device in a large clinic with multiple radiologists (servers) and presence of interrupting cases with the highest priority that overrides all cases triaged by CADt. An interesting approach using data-driven, feature-based priority queueing was proposed by [14], which minimizes waiting costs as part of the cost function to obtain the optimal number of priority queues when designing a queueing system in a radiology department. However, currently FDA-cleared CADt devices do not optimize the entire clinical workflows. They target only a single time-sensitive condition for prioritization, and our goal is to estimate the time savings that they achieve for patients with that condition with respect to the existing workflow. To our knowledge, no previous analyses theoretically predict the reduction in waiting time due to the deployment of a CADt device into an existing clinical workflow. Therefore, this work fills this gap by developing a queueing-theory-based evaluation framework to characterize the time-saving performance of a CADt device in clinical settings.

**Fig. 1:**
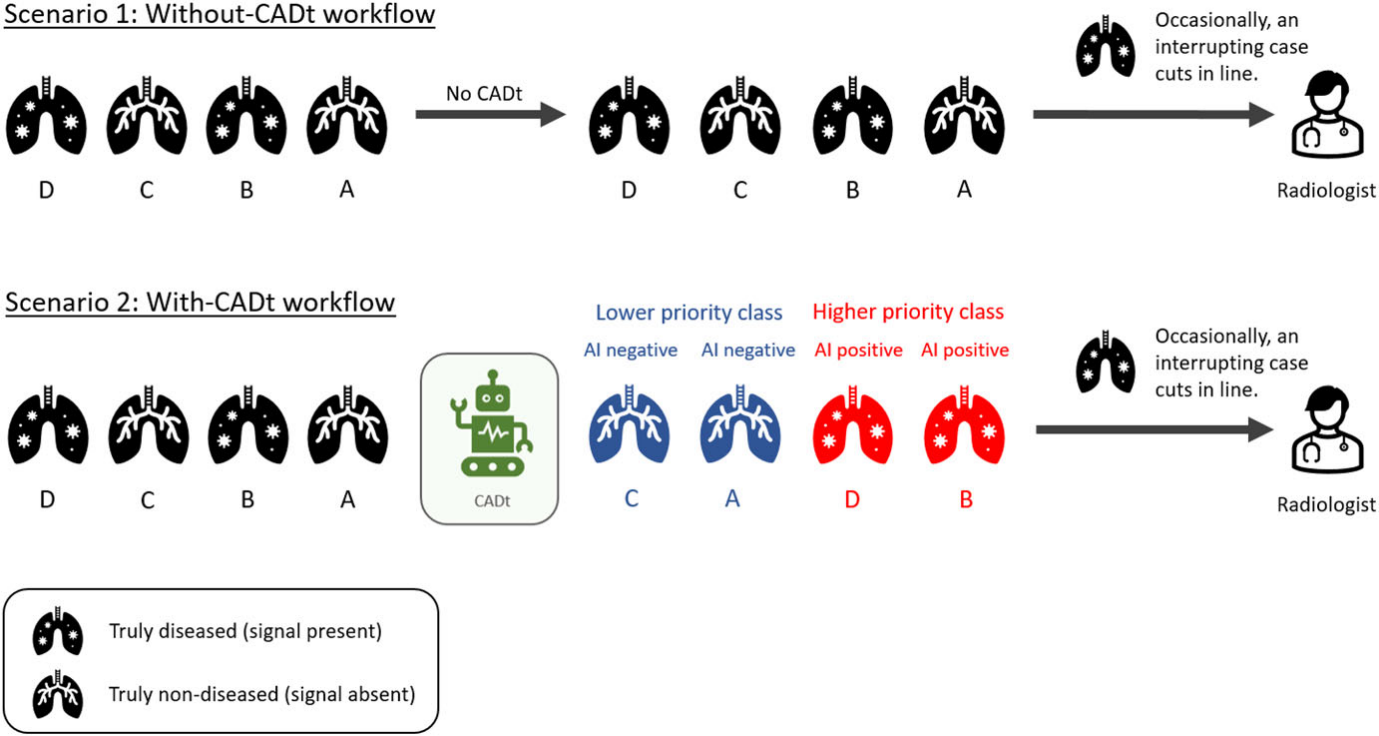
Radiologist workflows without and with a CADt device. *Top*: the without-CADt scenario in which patient images are reviewed in the order of their arrival. *Bottom*: the with-CADt workflow in which AI-positive patient images are reviewed first before the AI-negative images. In both scenarios, the radiologist may be interrupted by interrupting cases. All cartoon icons are adopted from Microsoft PowerPoint application

**Fig. 2:**
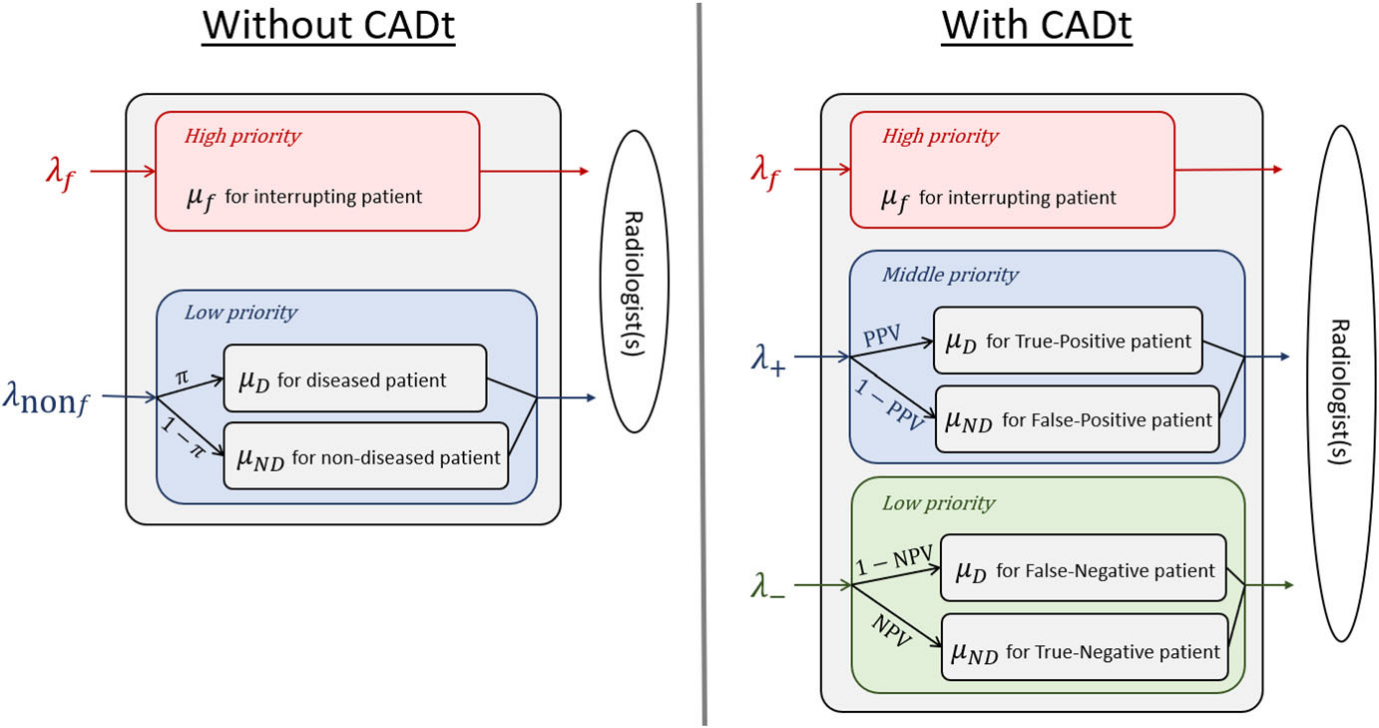
Priority classes in two scenarios. *Left*: Without-CADt scenario has two priority classes. Interrupting patient images with an arrival rate of $\lambda _f$ have a higher priority, whereas non-interrupting patient images ($\lambda _{\text {non}f}$) have a lower priority. Within the non-interrupting priority class is a mix of diseased and non-diseased patient images. *Right*: The with-CADt scenario has three priority classes. Interrupting patient images with an arrival rate of $\lambda _f$ have the highest priority, AI-positive patient images ($\lambda _+$) have a middle priority, and AI-negative patient images ($\lambda _-$) have the lowest priority. Within the AI-positive class, there is a mix of diseased patient images (i.e., true-positive) and non-diseased patient images (i.e., false-positive). Similarly, AI-negative cases consist of diseased (false-negative) patient images and non-diseased (true-negative) patient images. The parameters associated with the arrows represent the conditional probabilities that a patient case belongs to a subgroup. For example, in the without CADt scenario, $\pi$ represents the probability that a non-interrupting case belongs to the diseased subgroup. See Sect. 2.1 for the definitions of the parameters

Figure 1 illustrates the radiologist workflows without and with a CADt device being used. In the standard of care without a CADt device, patient images in the reading list are reviewed by a radiologist in the order of their arrivals to the list. In the context of queuing theory, our servers are radiologists, and our customers are patient images. Occasionally, the radiologist may be interrupted by an interrupting case, for example, when a physician requests an immediate review of a specific patient image. To distinguish these interrupting cases from those in the reading queue, we call the images in the reading list ”non-interrupting.” Therefore, a without-CADt scenario is a two-priority-class queueing system: interrupting and non-interrupting priority classes. If a CADt device is included in the workflow, the device only analyzes non-interrupting patient images. Cases labeled as AI-positive are either flagged or moved up in a radiologist’s reading list, giving them higher priority, and the radiologist will review those cases before reviewing any AI-negative patient images. Just like the without-CADt scenario, the radiologist may be interrupted by interrupting cases, which always have the highest priority over other images. Overall, as summarized in Fig. 2, without a CADt, we have a queue with two priority classes, and we have a queue with three priority classes in a with-CADt scenario.

In this work, we apply queueing theory to characterize the wait-time savings due to the use of a CADt for prioritization and demonstrate its use in a radiology clinic setting. In Sect. 2, we first define commonly used queueing parameters in the context of a radiologist workflow (Sect. 2.1). We formulate two queueing models in Sect. 2.2 from which the mean wait-times of patients in different priority classes are calculated. We then define a time-saving metric in Sect. 2.3. To provide the confidence intervals of the waiting time and to verify the theoretical results, we developed a simulation software to simulate radiologist workflows with and without a CADt device (Sect. 2.4). Last, we present the results of each workflow parameter to the time-saving performance of a CADt device (Sect. 3) and discuss limitations of our modeling in Sect. 4. We would like to emphasize that our contribution lies in the application of existing queueing theory to evaluate the time-saving of an AI-enabled triage devices with its false-positive and false-negative rates.

Although this work is motivated by the evaluation of CADt devices, this evaluation framework can be used to describe other systems designed to improve time-savings (or delay) to costumers triaged (or de-prioritized) by a classifier with type I (false positive) and type II (false negative) errors. Examples might be an AI algorithm to prioritize cybersecurity threats (deciding which one should be handled first) or an image processing AI that attempts to automatically identify customers with fewer items and move them to a shorter queue.

## Methods

### Parameters

Before applying queueing theory, a few parameters are defined to describe the clinical setting.

- *f* is the fraction of interrupting patient images with respect to all patient images. An example of such an interrupting case would be a very urgent patient case that an ER physician requests an immediate review by the radiologist.
- The disease prevalence $\pi$ is defined as the proportion of patients who have a time-critical condition within the non-interrupting patient population, i.e.,
  1 $$\begin{aligned} \pi = \frac{\text {Number of diseased, non-interrupting cases}}{\text {Number of non-interrupting cases}}. \end{aligned}$$
- CADt diagnostic performance is defined by its sensitivity ($\text {Se}$) and specificity ($\text {Sp}$), which are also defined within the non-interrupting patient images, i.e.,
  $$\begin{aligned} \text {Se} = \frac{\text {Number of AI-positive, diseased, non-interrupting cases}}{\text {Number of diseased, non-interrupting cases}}, \end{aligned}$$
  and
  $$\begin{aligned} \text {Sp} = \frac{\text {Number of AI-negative, non-diseased, non-interrupting cases}}{\text {Number of non-diseased, non-interrupting cases}}. \end{aligned}$$
- $\lambda$ is the Poisson arrival rate of all patient images. Patient images can be divided into subgroups based on priority classes (interrupting, non-interrupting, AI-positive, AI-negative). Each subgroup *i* has a Poisson arrival rate $\lambda _i = p_i\lambda$, where $p_i$ is the fraction of image subgroup *i* with respect to all patient images. In the without-CADt scenario, the arrival rates for the two priority classes (interrupting and non-interrupting) are given by
  2 $$\begin{aligned} \lambda _f = f\lambda \end{aligned}$$
  and
  3 $$\begin{aligned} \lambda _{\text {non}f} = (1-f)\lambda . \end{aligned}$$
  With a CADt device, there are three priority classes: interrupting, AI-positive, and AI-negative. The arrival rate of the interrupting class is the same as Eq. 2, but the arrival rates for AI-positive and AI-negative classes depend on the disease prevalence $\pi$ and the diagnostic performance of the CADt ($\text {Se}$ and $\text {Sp}$).
  4 $$\begin{aligned} \lambda _{+}= &   \big [\pi \text {Se} + (1-\pi )(1-\text {Sp})\big ]\lambda \end{aligned}$$
  5 $$\begin{aligned} \lambda _{-}= &   \big [\pi (1-\text {Se}) + (1-\pi )\text {Sp}\big ]\lambda . \end{aligned}$$
- $N_{\text {rad}}$ is the number of radiologists on-site. Typically, a clinic has at least one radiologist at all times. For a larger hospital, multiple radiologists may be available during the day.
- The radiologist’s reading rates are denoted by $\mu$’s. For interrupting (highest-priority) cases, the reading time is assumed to be exponentially distributed with an average reading rate $\mu _f$. For a non-interrupting image, the average reading rate depends on the radiologist’s diagnosis, i.e., $\mu _D$ if diseased image or $\mu _{ND}$ if non-diseased image. In the without-CADt scenario, the reading time of the non-interrupting (lower-priority) cases follows a hyperexponential distribution where the mean $(1/\mu _{\text {non}f})$ is determined by the mean reading rates of the two subgroups and the probability of disease prevalence $\pi$.
  6 $$\begin{aligned} \frac{1}{\mu _{\text {non}f}} = \frac{\pi }{\mu _D} + \frac{1-\pi }{\mu _{ND}}. \end{aligned}$$
  In the with-CADt scenario, the average reading rates for AI-positive (middle-priority) and AI-negative (lowest-priority) classes are denoted by $\mu _+$ and $\mu _-$, respectively. The AI-positive group consists of true-positive (TP) and false-positive (FP) patients, and the probability that an AI-positive case is a TP is defined by the positive predictive value (PPV). Hence,
  7 $$\begin{aligned} \frac{1}{\mu _+} = \frac{\text {PPV}}{\mu _D} + \frac{1-\text {PPV}}{\mu _{ND}}. \end{aligned}$$
  Similarly, the average AI-negative reading rate is given by
  8 $$\begin{aligned} \frac{1}{\mu _-} = \frac{1-\text {NPV}}{\mu _D} + \frac{\text {NPV}}{\mu _{ND}}, \end{aligned}$$
  where $\text {NPV}$ is the probability that an AI-negative case is a true-negative (TN). For this analysis, reading times of a radiologist for the diseased and non-diseased subgroups are assumed to be exponential. While this assumption needs further confirmation from clinical data, we start with the simplest exponential assumption as the first step.
- $\rho$ is the traffic intensity defined as $\rho = \lambda /\mu N_{\text {rad}}$. By definition, $\rho$ compares how fast an new image arrives and how fast the radiologists read an image. In this work, we focus on a normally operated system, where $\rho$ ranges from 0 (indicative of a fairly idle system) to 1 (indicative of a close-to-capacity system).
- With regard to the queueing discipline, we assume a preemptive-resume priority. Under this assumption, when a high priority image arrives, and if a radiologist is reviewing an image of low priority, the review of this low-priority image would be interrupted immediately. Once there is no more images of high priority in the queue, the radiologist will resume the review of the previously interrupted case. It is noted that, although in reality some radiologists may prefer finishing up the current image in hand when a CADt device flags a case, CADt devices are evaluated assuming that a radiologist reads the flagged cases immediately, maximizing the time-saving benefits to the diseased patient images.

### Radiologist workflow models

We consider two radiologist workflow models:

- Model 1: $$\mu _D = \mu _{ND}$$ and $$N_{\text {rad}} > 1$$
- Model 2: $$\mu _D \ne \mu _{ND}$$ and $$N_{\text {rad}} = 1$$

The major difference between Models 1 and 2 is that the reading rates between diseased and non-diseased cases are the same in Model 1 but can be different in Model 2. Clinically, observational studies have shown that the average reading time depends on the imaging modalities [3–5]. However, whether the mean reading time for diseased cases is the same as that for non-diseased cases given the same imaging modality is not well studied. It is hypothesized that a radiologist may take longer to complete a diseased case (e.g., documenting the findings, contacting physicians for an immediate follow-up, etc.), whereas a non-diseased case can be processed faster without the subsequent tasks. Therefore, we investigate both workflow models in this work.

The technical difficulty in Model 2 lies in the fact that the system has to remember the disease state of the case that was previously interrupted. For example, in the without-CADt scenario, when a diseased patient image was interrupted by an incoming interrupting image, the system should remember that it must come back to the diseased case (not a non-diseased case). Therefore, we extended RDR technique to add this "memory" in the transition matrices for Model 2.

For each model, two scenarios are examined: one assumes a without-CADt scenario and the other assumes the use of a CADt device. Each scenario has a set of *states* that keeps track of the numbers of patient images in different priority classes. For example, $n_j$ is the number of patient images in the system in the priority class *j*; the number of images in the system also includes those currently being reviewed by radiologists. The transition rates among states form a stochastic Markov chain matrix, from which the matrix geometric method is applied to calculate the set of state probabilities [15]. Given the multi-server, multi-class nature of our workflow models, the full transition rate matrix is multi-dimensional. To simplify the calculation, we apply the recursive dimensionality reduction (RDR) method proposed by [6], which disentangles the multi-dimensional matrix by truncating states of higher dimensions and decomposing the matrix into per-priority matrices. Using the RDR method, we calculate the state probability for each priority class $p_j$, where *j* is the priority class. With $p_j$, the mean waiting time for patient images in the priority class can be calculated by the following steps.

1. Calculate the average number of patient images in the priority class in the system, $L_j$, from the state probability $p_j$. That is, $L_j =\sum ^{\infty }_{i=n_j} n_j p_j$.
2. Calculate the average response time, $W_j$, via Little’s Law [15], i.e., $W_j = L_j/\lambda _j$.
3. Calculate the average waiting time in the queue, $W_{q_j}$. Because $W_j$ is the sum of $W_{q_j}$, along with the mean radiologist’s reading time $1/\mu _j$ for patient images in that priority class, we have $W_{q_j} = W_j - 1/\mu _j$.

In summary, the mean waiting time of patient images in a priority class, $W_{q_j}$, is given by

9 $$\begin{aligned} W_{q_j} = \langle p_j\rangle /\lambda _j - 1/\mu _j. \end{aligned}$$

From Eq. 9, the mean waiting time for patient images in a given priority class can be calculated once the state probability $p_j$ is known. Therefore, in the following subsections, we focus on the calculation of the state probability for different priority classes in with- and without-CADt scenarios of the two radiologist workflow models. It should be noted that this work only focuses on the wait-time calculation for the non-interrupting, AI-positive, and AI-negative subgroups impacted by the CADt device. Although one can also calculate the wait-time of the interrupting subgroup, these images always cut in line, and hence, their wait-time will not be affected by the use of a CADt device in a preemptive setting. All matrices are provided in the appendix, and the calculation code is publicly available in GitHub [18].

#### Model 1

Model 1 assumes that the reading rate of a radiologist is the same for diseased and non-diseased subgroups. Because $\mu _D = \mu _{ND}$, the reading rates for the non-interrupting subgroups (without-CADt), as well as the AI-positive and AI-negative subgroups (with CADt), are the same; $\mu _+ = \mu _- = \mu _{\text {non}f}$. This simplifies the transition matrices which can be generalized to any number of radiologists; $N_{\text {rad}} > 1$. As an illustration, the following discussion focus on $N_{\text {rad}} = 2$, which follows the example in [6].

*Without-CADt scenario*

In the standard of care without a CADt device, there are two priority classes: interrupting and non-interrupting.

**Fig. 3:**
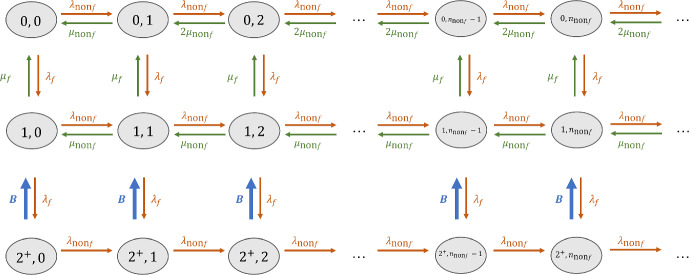
The RDR-truncated transition diagram for non-interrupting patient images in Model 1 without a CADt device. The states $(n_f, n_{\text {non}f})$ are defined by the numbers of interrupting and non-interrupting cases in the system

The RDR-truncated transition diagram for the non-interrupting priority class is given in Fig. 3. Given two radiologists on-site, a non-interrupting image can exit the system only when there are less than two interrupting images in the system, i.e., $n_f < 2$, and hence the truncation of states starts when $n_f = 2$. When $n_f = 0$, both radiologists are available for non-interrupting patient images. Thus, the first row has a leaving rate $2\mu _{\text {non}f}$, except the transition from (0, 1) to (0, 0) when only one radiologist has work to do. When $n_f = 1$ (the second row), only one of the two radiologists is available to review a non-interrupting case, resulting in a departure rate of $1\mu _{\text {non}f}$. When $n_f \ge 2$, both radiologists are busy handling interrupting cases. Since no radiologist is available for non-interrupting images, their departure rate is 0, and no non-interrupting images can leave the system.

**Fig. 4:**
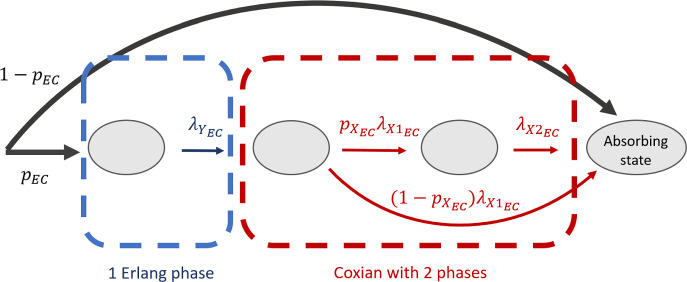
An Erlang–Coxian (EC) distribution with one Erlang phase and two Coxian phases. See [11] for the closed form solutions to calculate these parameters

To approximate the transition rate ${\textbf {B}}$ in Fig. 3, a simplified Erlang–Coxian (EC) approximation known as two-phase Coxian is applied (the red box in Fig. 4). An EC distribution is a class of phase type distribution that consists of any number of Erlang phases followed by a two-phase Coxian. [11] proved that a distribution of any shapes can be approximated by an EC distribution up to the first three moments ($\mu _1$, $\mu _2$, $\mu _3$) and provided a closed form solution to solve for the six parameters involved. The number of Erlang phases needed depends on where the two relative moments ($\mu _2/\mu _1$, $\mu _3/\mu _1$) lie in a two-dimensional space. The busy periods discussed in [6] only require two Coxian phases without any Erlang phases. Hence, $p_{_{\text {EC}}}$, $N_{_{\text {EC}}}$, and $\lambda _{Y_{\text {EC}}}$ in Fig. 4 are 1, 2, and 0, respectively. The non-exponential transition ${\textbf {B}}$ is explicitly expressed in terms of the approximated exponential transition rates *t*, where

10 $$\begin{aligned} {\begin{matrix} t_1 &  = (1-p_{X_{\text {EC}}})\lambda _{X1_{\text {EC}}} \\ t_{12} & = p_{X_{\text {EC}}}\lambda _{X1_{\text {EC}}} \\ t_2 & = \lambda _{X2_{\text {EC}}}. \end{matrix}} \end{aligned}$$

With the *t* parameters, the transition rate matrix $M_{1_{\text {noCADt}}}$ for Fig. 3 can be found in Appendix (Eq. 16). From the transition rate matrix, the state probability $p_{\text {non}f}$ is determined, and the mean waiting time per non-interrupting patient image $W_{q_{\text {non}f}}$ is given by Eq. 9.

*With-CADt scenario*

With-CADt scenario has three priority classes: interrupting (highest priority), AI-positive (middle priority), and AI-negative (lowest priority). The calculations of state probabilities for AI-positive and AI-negative subgroups are separated using the RDR method.

Because of the preemptive-resume queueing discipline, the AI-positive subgroup is not affected by the AI-negative images at all. Hence, the queueing system for the AI-positive subgroup can be described by two priority classes: the interrupting and AI-positive classes. This can be equivalently viewed as the non-interrupting subgroup in the without-CADt scenario. Therefore, by replacing $\lambda _{\text {non}f}$ with $\lambda _+$ and $\mu _{\text {non}f}$ with $\mu _+$ in Fig. 3 and Eq. 16, the state probability for the AI-positive subgroup $p_+$ can be computed. And the mean waiting time per an AI-positive patient image $W_{q_+}$ is given by Eq. 9.

**Fig. 5:**
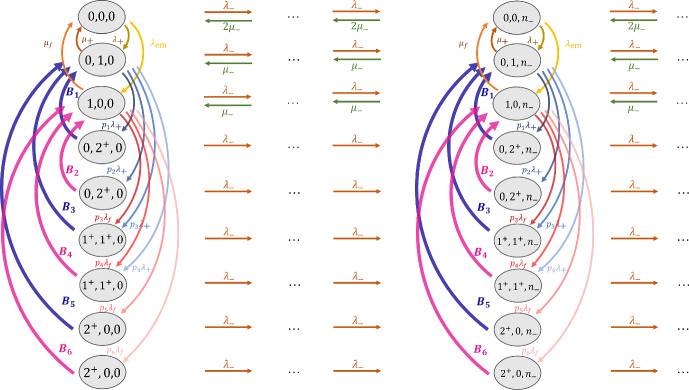
The RDR-truncated transition diagram for AI-negative patient images in Model 1 with a CADt device. The state is defined as $(n_f, n_+, n_-)$, and states with $n_f + n_+ \ge 2$ are truncated. A total of 6 busy periods are identified. Each busy period *i* has a transition rate ${\textbf {B}}_i$ along with a conditional probability that it ends at a certain state given that it starts with a particular state

For the calculation of the AI-negative class, we cannot ignore the presence of AI-positive cases, and a state is defined as $(n_f, n_+, n_-)$. As discussed in [6], we need to consider busy periods, i.e., the time duration in which all the radiologists on-site are too busy for AI-negative patient images. With two radiologists, a busy period may start from one of the three situations: when there are two interrupting cases, when there are one interrupting and one AI-positive case, or when there are two AI-positive cases. On the other hand, the busy period ends when there is only one interrupting case or one AI-positive case such that one of the two radiologists is available for the AI-negative case. This results in a total number of six busy periods:

- $$B_1$$: ($$0^+$$, $$2^+$$, $$n_-$$) $$\rightarrow $$ (0, 1, $$n_-$$)
- $$B_2$$: ($$0^+$$, $$2^+$$, $$n_-$$) $$\rightarrow $$ (1, 0, $$n_-$$)
- $$B_3$$: ($$1^+$$, $$1^+$$, $$n_-$$) $$\rightarrow $$ (0, 1, $$n_-$$)
- $$B_4$$: ($$1^+$$, $$1^+$$, $$n_-$$) $$\rightarrow $$ (1, 0, $$n_-$$)
- $$B_5$$: ($$2^+$$, $$0^+$$, $$n_-$$) $$\rightarrow $$ (0, 1, $$n_-$$)
- $$B_6$$: ($$2^+$$, $$0^+$$, $$n_-$$) $$\rightarrow $$ (1, 0, $$n_-$$)

Please note that $0^+$ indicates that, during a busy period, there could be some high priority patient cases. However, the end of the busy period can only have one high priority case (either interrupting or AI-positive).

Figure 5 shows the RDR-truncated transition diagram for the AI-negative subgroup. States $(0, 2^+, n_-)$, $(1^+, 1^+, n_-)$, and $(2^+, 0, n_-)$ are duplicated because their corresponding arrival rates also depends on the probabilities that the busy period ends at a particular state, i.e., either $(0, 1, n_-)$ or $(1, 0, n_-)$. For example, $p_1$ denotes the conditional probability that the busy period ends at $(0, 1^+, n_-)$ given that it starts at $(0, 2^+, n_-)$.

Before solving for Fig. 5, one must compute the conditional probability and the first three moments of each busy period, from which the transition rates of ${\textbf {B}}$s can be approximated. The calculation is discussed in Appendix of [6] via inter-level passage times; the formulae for this specific workflow model is provided in this Appendix (Eq. 18).

An EC approximation is performed for each busy period (Fig. 4). $B_1$, $B_3$, $B_4$, and $B_6$ can be approximated with only two Coxian phases, and their *t* parameters are calculated via Eq. 10). However, for $B_2$ and $B_5$, an extra Erlang phase is needed, resulting in two extra parameters $t_0$ and $t_{01}$.

11 $$\begin{aligned} {\begin{matrix} t_0 &  = (1-p_{_{\text {EC}}})\lambda _{Y_{\text {EC}}} \\ t_{01} & = p_{_{\text {EC}}}\lambda _{Y_{\text {EC}}} \\ t_1 &  = (1-p_{X_{\text {EC}}})\lambda _{X1_{\text {EC}}} \\ t_{12} & = p_{X_{\text {EC}}}\lambda _{X1_{\text {EC}}} \\ t_2 & = \lambda _{X2}. \end{matrix}} \end{aligned}$$

Once all six busy periods are approximated, the transition rate matrix for the AI-negative class is constructed from Fig. 5 (Eq. 20). The corresponding state probability $p_-$ can be solved by the matrix geometric method. And, the mean waiting time per AI-negative patient image $W_{q_-}$ can be calculated via Eq. 9.

For $N_{\text {rad}} \ge 3$, the same approach can be applied. However, as the number of busy periods increases, the transition rate matrix grows in size drastically, especially if more Erlang phases are needed for the busy period approximation. While we do not present the transition rate matrices in this paper for $N_{\text {rad}} \ge 3$, matrix construction and mean wait-time calculation for Model 1 are generalized to any $N_{\text {rad}}$ in our publicly available software tool [18].

#### Model 2

Model 2 differentiates the radiologist’s reading rate between the diseased and non-diseased subgroups. Because $\mu _D \ne \mu _{ND}$, the reading rates for non-interrupting, AI-positive, and AI-negative subgroups depend on disease prevalence $\pi$, positive predictive value $\text {PPV}$, and negative predictive value $\text {NPV}$ (Eqs. 6-8).

*Without-CADt scenario*

**Fig. 6:**
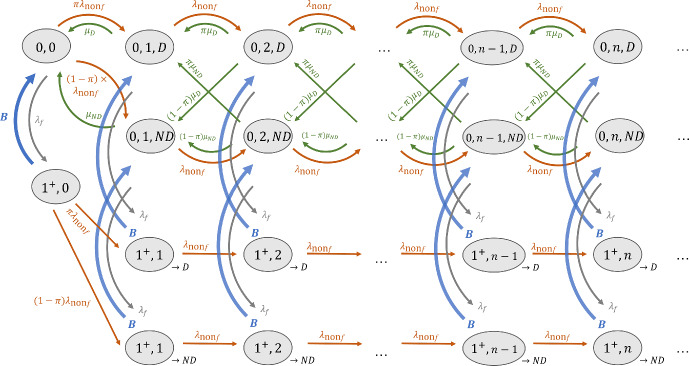
The RDR-truncated transition diagram for non-interrupting patient images in Model 2 in a without-CADt scenario. The state is defined by the number of interrupting patient images (either 0 or $1^+$), number of non-interrupting patient images *n*, and the disease status of case that the one radiologist is reviewing (either *D* for diseased or *ND* for non-diseased). The “$\rightarrow D$" and “$\rightarrow ND$" in the truncated states keep track of the disease status of the interrupted case

The without-CADt scenario has two priority classes: interrupting and non-interrupting patient images. Within the non-interrupting class, two groups of patient images (diseased and non-diseased) are reviewed in a first-in-first-out basis. The corresponding transition diagram is shown in Fig. 6, where a state keeps track of $n_f$ and $n_{\text {non}f}$. Because of the different reading rates between the diseased and non-diseased subgroups, the state must also keep track of the disease status of the image that the radiologist is reviewing. Therefore, the state is defined as $(n_f, n_{\text {non}f}, i)$, where *i* is either *D* (i.e., the radiologist is working on a diseased patient image) or *ND* (i.e., the radiologist is working on a non-diseased image). Furthermore, to make sure that the radiologist resumes the previously interrupted case, the state needs to “remember” how the busy period started. For example, if the radiologist reading a diseased image is interrupted by the arrival of an interrupting image, i.e., $(0, n, D) \rightarrow (1^+, n, D)$, the state must go back to (0, *n*, *D*) when the busy period is over and not to (0, *n*, *ND*). This property is guaranteed by having two sets of truncated states: $(1^+, n)_{\rightarrow D}$ which can only interact with (0, *n*, *D*) and $(1^+, n)_{\rightarrow ND}$ which can only interact with (0, *n*, *ND*). Note that although Fig. 6 has two busy periods per column (one for “$\rightarrow D$” and the other for “$\rightarrow ND$”), they both describe the same transition time when at least one interrupting image is in the system. Therefore, only one unique set of *t* parameters is calculated to approximate both busy periods.

The corresponding transition rate matrix of Fig. 6 is given in Eq. 23. The corresponding state probability $p_{\text {non}f}$ is solved by the matrix geometric method, and the mean waiting time per AI-negative patient image $W_{q_{\text {non}f}}$ can be calculated via Eq. 9.

*With-CADt scenario*

With a CADt device, the calculation for AI-positive (middle priority) and AI-negative (lowest priority) subgroups is separated using the RDR method.

Because AI-positive patient images are not impacted by AI-negative cases, the interrupting and AI-positive subgroups form a two-priority-class queueing system. The transition rate matrix in Eq. 23 from Fig. 6 can be reused to analyze the queueing of the AI-positive patient images. By replacing $\lambda _{\text {non}f}$ by $\lambda _+$, $\mu _{\text {non}f}$ by $\mu _+$, and $\pi$ by $\text {PPV}$, the state probability for the AI-positive subgroup $p_+$ is calculated via standard matrix geometric method. The mean waiting time per AI-positive patient image $W_{q_+}$ is then given by Eq. 9.

**Fig. 7:**
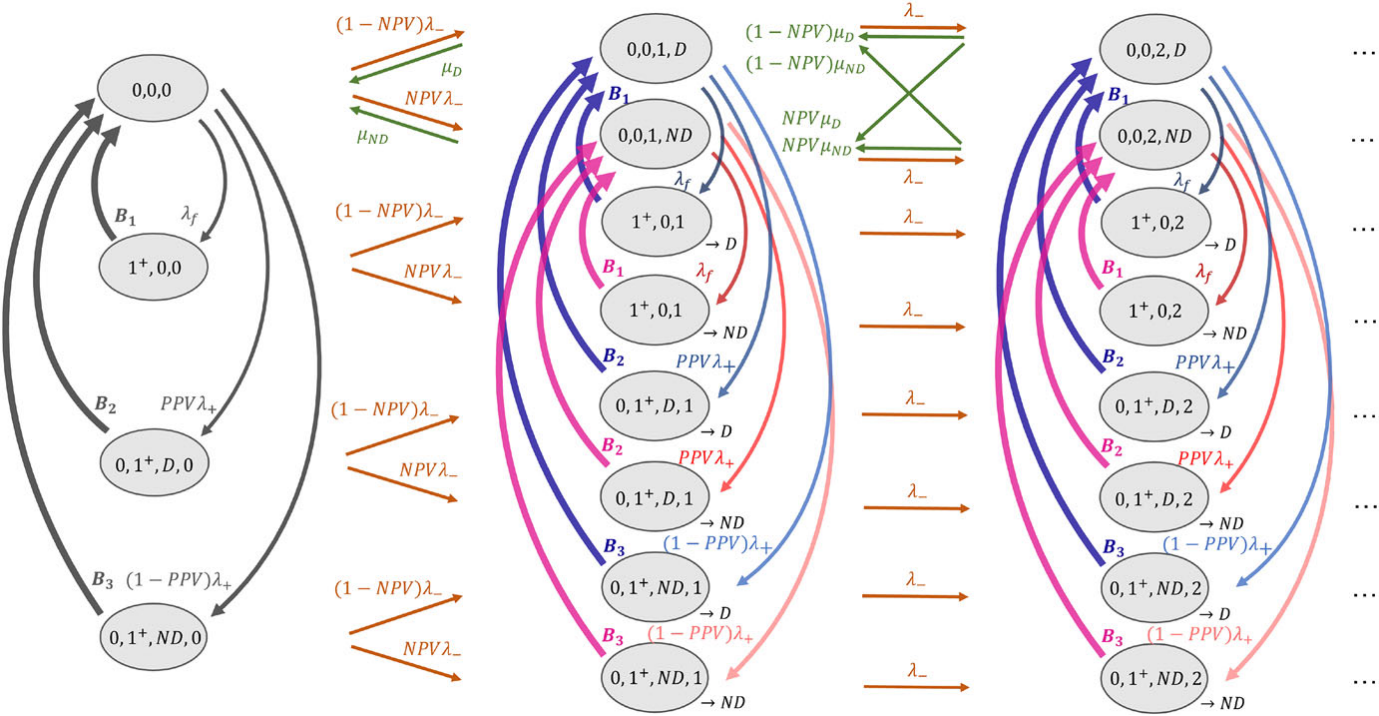
The RDR-truncated transition diagram for AI-negative subgroup in Model 2 with a CADt device. State is defined as $(n_f, n_+, i, n_-, j)$, where *i* (or *j*) indicates the disease status of the AI-positive (or AI-negative) case the radiologist is reading

For the AI-negative class, the full definition of state $(n_f, n_+, i, n_-, j)$. *i* is either *D* or *ND*, indicating whether the radiologist is working on a diseased, AI-positive (i.e., true-positive) case or a non-diseased, AI-positive (i.e., false-positive) case, respectively. Similarly, the disease status of an AI-negative case that the radiologist is reading is represented by the *j* which is either *D* or *ND*. Because we only have one radiologist, *i* and *j* cannot appear simultaneously; the one radiologist can only handle an AI-positive or AI-negative case but not both at the same time.

Figure 7 shows the RDR-truncated transition diagram for the AI-negative subgroup. There are three unique busy periods with the corresponding transition rates $B_i$:

- $$B_1$$: $$(1^+, 0^+, n_-)_{\rightarrow j}$$ $$\rightarrow $$ (0, 0, $$n_-$$, *j*)
- $$B_2$$: $$(0^+, 1^+, D, n_-)_{\rightarrow j}$$ $$\rightarrow $$ (0, 0, $$n_-$$, *j*)
- $$B_3$$: $$(0^+, 1^+, ND, n_-)_{\rightarrow j}$$ $$\rightarrow $$ (0, 0, $$n_-$$, *j*)

For each busy period, the truncated state is duplicated with either “$\rightarrow D$" or “$\rightarrow ND$" such that the system can return to the state with the correct disease status *j* when the busy period is over.

For each unique busy period, its conditional probability and first three moments are determined from the transition probability matrix (Eq. 25). Each unique busy period has a set of *t* parameters (Eq. 10) from the EC approximation. With the approximated busy period transitions, a transition rate matrix can be constructed for the AI-negative subgroup (Eq. 27). The state probability $p_-$ is then solved, and the mean waiting time per AI-negative patient image $W_{q_-}$ can be calculated via Eq. 9.

### Wait-time-saving performance metric

We define a metric to quantitatively assess the wait-time-savings of a given CADt device. Both theoretical and simulation approaches output the mean waiting time per diseased patient image $W_{\text {D}}$ in both with- and without-CADt scenarios.

Without a CADt device, since the arrival process is random, the average waiting time per non-interrupting patient image $W^{\text {no-CADt}}_{\text {non}f}$ is the same as $W^{\text {no-CADt}}_{\text {D}}$, i.e.,

12 $$\begin{aligned} W^{\text {no-CADt}}_{\text {D}} = W_{q_{\text {non}f}}. \end{aligned}$$

The calculation of $W_{q_{\text {non}f}}$ for Model 1 and 2 are discussed in the corresponding Without-CADt scenario subsections, respectively. When a CADt device is included in the workflow, the average waiting time per diseased and non-diseased patient images are no longer the same because the diseased images are more likely to be prioritized by the CADt. To calculate $W^{\text {CADt}}_{\text {D}}$, we first compute the average waiting time per AI-positive ($W^{\text {CADt}}_{+} = W_{q_+}$) and per AI-negative ($W^{\text {CADt}}_{-} = W_{q_-}$) patient image based on the mathematical frameworks discussed in Section 2.2. By definition, the average waiting time for the diseased subgroup $W^{\text {CADt}}_{\text {D}}$ is

$$\begin{aligned} W^{\text {CADt}}_{\text {D}} \equiv \frac{\text {Total waiting time from all diseased patient images}}{\text {Number of diseased patient images}}. \end{aligned}$$

Note that the total waiting time from all diseased patients is the sum of the total waiting time from the true-positive (TP) subgroup and that from the false-negative (FN) subgroup. Let $N_{\text {TP}}$, $N_{\text {FN}}$, and $N_{\text {D}}$ be the number of TP patient images, that of FN patient images, and that of diseased images. $W^{\text {CADt}}_{\text {D}}$ can be rewritten as

$$\begin{aligned} W^{\text {CADt}}_{\text {D}} = \frac{W^{\text {CADt}}_{+} \times N_{\text {TP}} + W^{\text {CADt}}_{-} \times N_{\text {FN}}}{N_{\text {D}}}. \end{aligned}$$

Because $N_{\text {TP}}/N_{\text {D}}$ and $N_{\text {FN}}/N_{\text {D}}$ are, by definition, $\text {Se}$ and $1-\text {Se}$, we have

13 $$\begin{aligned} W^{\text {CADt}}_{\text {D}} = W^{\text {CADt}}_{+} \times \text {Se} + W^{\text {CADt}}_{-} \times (1-\text {Se}). \end{aligned}$$

The calculation of $W_{q_+}$ and $W_{q_-}$ for Models 1 and 2 is discussed in the corresponding With-CADt scenario subsections, respectively.

To quantify the wait-time-savings of a CADt device for diseased patient images, we define a time performance metric $\delta W_\text {D}$ as the difference in mean waiting time per diseased image in the with-CADt and that in the without-CADt scenario:

14 $$\begin{aligned} \delta W_{\text {D}} \equiv W^{\text {CADt}}_{\text {D}} - W^{\text {no-CADt}}_{\text {D}}. \end{aligned}$$

It should be noted that, besides the explicit dependence on AI sensitivity in Eq. 13, $\delta W_{\text {D}}$ also depends on AI specificity and all the clinical factors in the calculation of $W^{\text {CADt}}_{+}$, $W^{\text {CADt}}_{-}$, and $W^{\text {no-CADt}}_{\text {non}f}$.

Based on its definition, a negative $\delta W_{\text {D}}$ implies that, on average, a diseased patient image is reviewed earlier when the CADt device is included in the workflow than when it is not. The more negative $\delta W_{\text {D}}$ is, the more time is saved, and the more effective the CADt device is. If $\delta W_{\text {D}} = 0$, the presence of CADt device does not bring any benefit for the diseased patient images. If $\delta W_{\text {D}}$ is positive, the review of a diseased patient image is delayed on average, and the CADt device brings more risks than benefits to the diseased subgroup.

An alternative metric would be the ratio between the two scenarios $W^{\text {CADt}}_{\text {D}}/W^{\text {noCADt}}_{\text {D}}$. However, we found that the difference is more intuitive in the clinical context because it better indicates the potential magnitude of the impact on patient outcome. As an example, our evaluation framework predicts a 10-minute saving for LVO stroke due to a CADt, with a corresponding 3 times effect compared to the without-CADt workflow. Clinicians have a better clinical sense for the 10-minute savings (which could be a substantial outcome difference for a stroke patient) compared to the 3-times-faster (which does not indicate as clearly the patient impact). Using the difference instead of ratio also allows researchers to compare results from our evaluation framework with empirical results from clinical studies such as [1, 7, 8, 20], in which performance of triage algorithm are generally reported as differences in time.

It should also be noted that the amount of time savings for other subgroups can be defined similarly. For example, for the non-diseased subgroup, the average waiting time per non-diseased patient image in the without-CADt scenario, $W^{\text {no-CADt}}_{\text {ND}}$, is

$$\begin{aligned} W^{\text {no-CADt}}_{\text {ND}} = W^{\text {no-CADt}}_{\text {non}f}. \end{aligned}$$

When the CADt device is included in the workflow, the average waiting time per non-diseased patient image, $W^{\text {CADt}}_{\text {ND}}$, becomes

$$\begin{aligned} W^{\text {CADt}}_{\text {ND}} = W^{\text {CADt}}_{+} \times (1-\text {Sp}) + W^{\text {CADt}}_{-} \times \text {Sp}, \end{aligned}$$

where the first and second terms correspond to the false-positive and true-negative subgroups, respectively. $\delta W_{\text {ND}}$ can then be defined to describe the average wait-time difference between the with-CADt and without-CADt scenarios for the non-diseased subgroup.

15 $$\begin{aligned} \delta W_{\text {ND}} \equiv W^{\text {CADt}}_{\text {ND}} - W^{\text {no-CADt}}_{\text {ND}} \end{aligned}$$

### Simulation

To verify our theoretical queueing approach, a Monte Carlo software was developed using Python to simulate the flow of patient images in a clinic with and without a CADt device. A workflow model is defined by a set of input parameters {*f*, $\pi$, $\rho$, $\mu _f$, $\mu _D$, and $\mu _{ND}$, $N_{\text {rad}}$, $\text {Se}$, and $\text {Sp}$}. It is noted that $\lambda$ is not explicitly stated but is calculated from $\rho$ and $\mu$s.

During a simulation, a new patient image entry is randomly generated with a timestamp that follows a Poisson distribution at an overall arrival rate of $\lambda$. Each patient image is randomly assigned with an interrupting status (interrupting or non-interrupting) based on the input interrupting fraction *f*. If the patient image is interrupting, a reading time is randomly generated from an exponential distribution with a reading rate of $\mu _f$. If the patient image is non-interrupting, a disease status (diseased or non-diseased) is randomly assigned based on the input disease prevalence $\pi$. The reading time for this non-interrupting patient image is also randomly drawn from an exponential distribution with a reading rate of either $\mu _D$ if it is diseased or $\mu _{ND}$ if it is non-diseased. Each non-interrupting patient image is also assigned with an AI-call status (positive or negative) based on its disease status and the input AI accuracy ($\text {Se}$ and $\text {Sp}$). The patient image is then simultaneously placed into two worlds: one with a CADt device and one without.

In a without-CADt world, the incoming patient image is either a high priority case (if it is interrupting) or a low-priority case (if non-interrupting). If the patient image is interrupting, the case is prioritized over all non-interrupting patient images in the system and is placed at the end of the interrupting-only queue. Otherwise, the patient image is non-interrupting and is placed at the end of the current reading queue. In time, when its turn comes, this patient image is read by one of the radiologists and is then removed from the queue. Two pieces of information are recorded for this simulated patient image. One is its waiting time defined as the difference between the time when the image enters the queue and when it leaves the queue. In addition, we also record the number of interrupting and non-interrupting patient images in the queue right before the arrival of the new patient image which is directly related to the state probability distribution.

Alternatively, this very same patient image is placed in the with-CADt world. This image has either a high priority (if interrupting), a middle priority (if AI-positive), or a low priority (if AI-negative). If the patient image is interrupting, the case is prioritized over all AI-positive and AI-negative patient images in the system and is placed at the end of the interrupting-only queue. If the patient image is AI-positive, the case is prioritized over all AI-negative images and is placed at the end of the queue consisting of only interrupting and AI-positive patient images. Otherwise, the patient image is AI-negative and is placed at the end of the current reading queue. The reading time for this patient image in the with-CADt world is identical to its reading time in the without-CADt world. However, due to the re-ordering by the CADt device, its waiting time in the with-CADt world may be different from that in the without-CADt world. For every patient image, the difference between the two waiting times in the two worlds is calculated to determine whether the use of the CADt device results in a time-saving or time delay for this image. In addition to its waiting time, the number of interrupting, AI-positive, and AI-negative patient images right before the arrival of the new patient image are also recorded.

To simulate a big enough sample size, a full simulation includes 200 simulations, each of which contains roughly 2,000 patients. From all simulations, the waiting times from all diseased patient images are histogrammed from which the mean value and the 95% confidence intervals are determined.

## Results

To demonstrate the agreement between the theoretical and simulation approaches, we assume a disease prevalence $\pi$ of 10%, an average reading time of 10 min for both diseased and non-diseased subgroups, and one radiologist on-site. For CADt diagnostic performance, we manually sifted through FDA clearance summaries for 82 FDA-cleared CADt devices and extracted the reported point estimates of sensitivity and specificity. A typical CADt performance is described by the average sensitivity (95%) and specificity (89%) across all CADt devices.

**Fig. 8:**
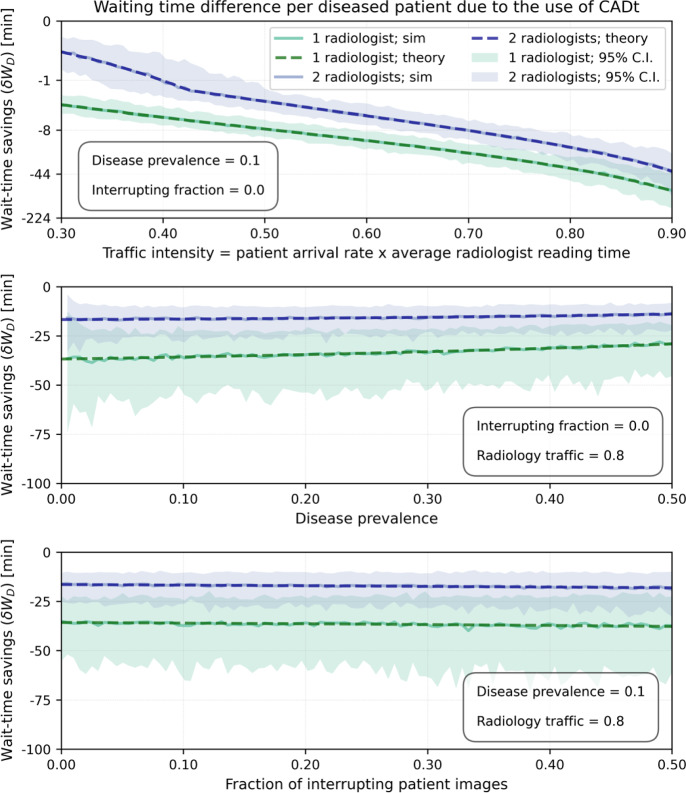
Amount of time saved per diseased patient image as a function of (top) traffic intensity, (middle) disease prevalence, and (bottom) interrupting fraction. Green and blue lines represent scenarios with one and two radiologists, respectively. Dashed lines are theoretical $\delta W_D$, and the solid lines represent the mean wait-time-savings from simulation. Shaded areas are the 95% confidence intervals (C.I.s) from simulation. The average reading time ($1/\mu _f$) for an interrupting image is set at 5 min, whereas the average reading time for the diseased ($1/\mu _D$) and non-diseased ($1/\mu _{ND}$) subgroups are both 10 min

The top plot in Fig. 8 shows the time saved per diseased patient image as a function of traffic intensity $\rho$ for one and two radiologists on-site without any interrupting patient images. The time savings from deployment of the CADt increases significantly with $\rho$ from about 2 min in a quiet, low-volume clinic (radiology traffic intensity of 0.3) to about an hour in a relatively busy clinic (radiology traffic intensity of 0.8). At $\rho$ of 0.8, disease prevalence has only a small impact on time savings (see middle plot in Fig. 8). As expected, the wait-time-savings from deployment of the CADt is more evident when only one radiologist is on-site. The bottom plot in Fig. 8 shows that interrupting images have minimal impact on wait-time-savings. That is, the amount of time saved per diseased image with and without any interrupting patient images ($f = 0$) is more-or-less the same at $f = 50$%.

**Fig. 9:**
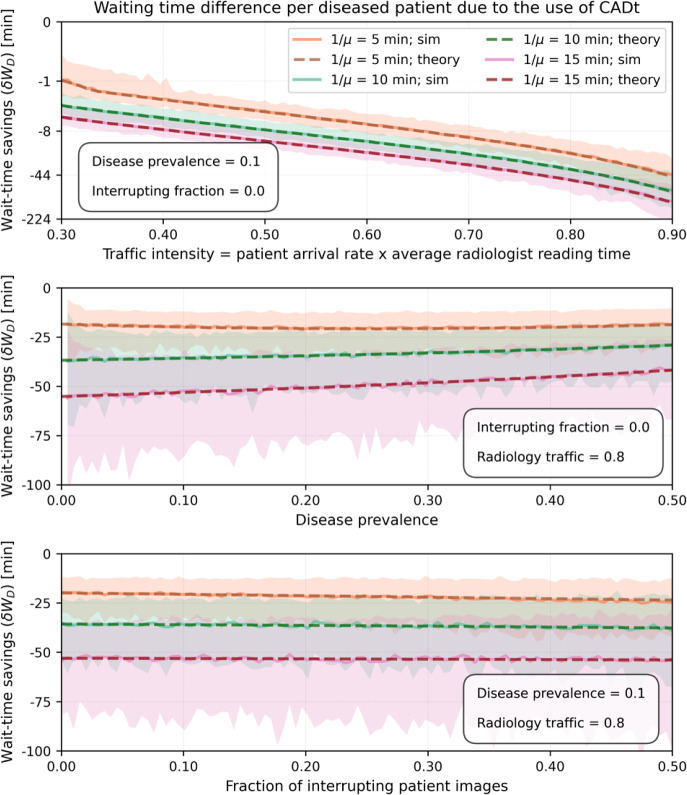
Amount of time saved per diseased patient as a function of (top) traffic intensity, (middle) disease prevalence, and (bottom) interrupting fraction. Only one radiologist is on-site, and its average reading times for interrupting ($1/\mu _f$) and diseased ($1/\mu _D$) patient images are set at 5 min and 10 min, respectively. The average reading time for non-diseased ($1/\mu _{ND}$) patient images varies between 5 min (orange), 10 min (green), and 15 min (red). Dashed lines are theoretical $\delta W_D$, and the solid lines represent the mean wait-time-savings from simulation. Shaded areas are the 95% confidence intervals (C.I.s) from simulation. Note that the green set of lines here is identical to that in Fig. 8

The effect of different radiologist’s reading rates for diseased and non-diseased subgroups is shown in Fig. 9. The overall dependence on traffic intensity, disease prevalence, and interrupting fraction is similar to that in Fig. 8. However, more time is saved for diseased patient images when $\mu _D > \mu _{ND}$, i.e., when a radiologist takes more time on average to read a non-diseased image than a diseased image.

**Fig. 10:**
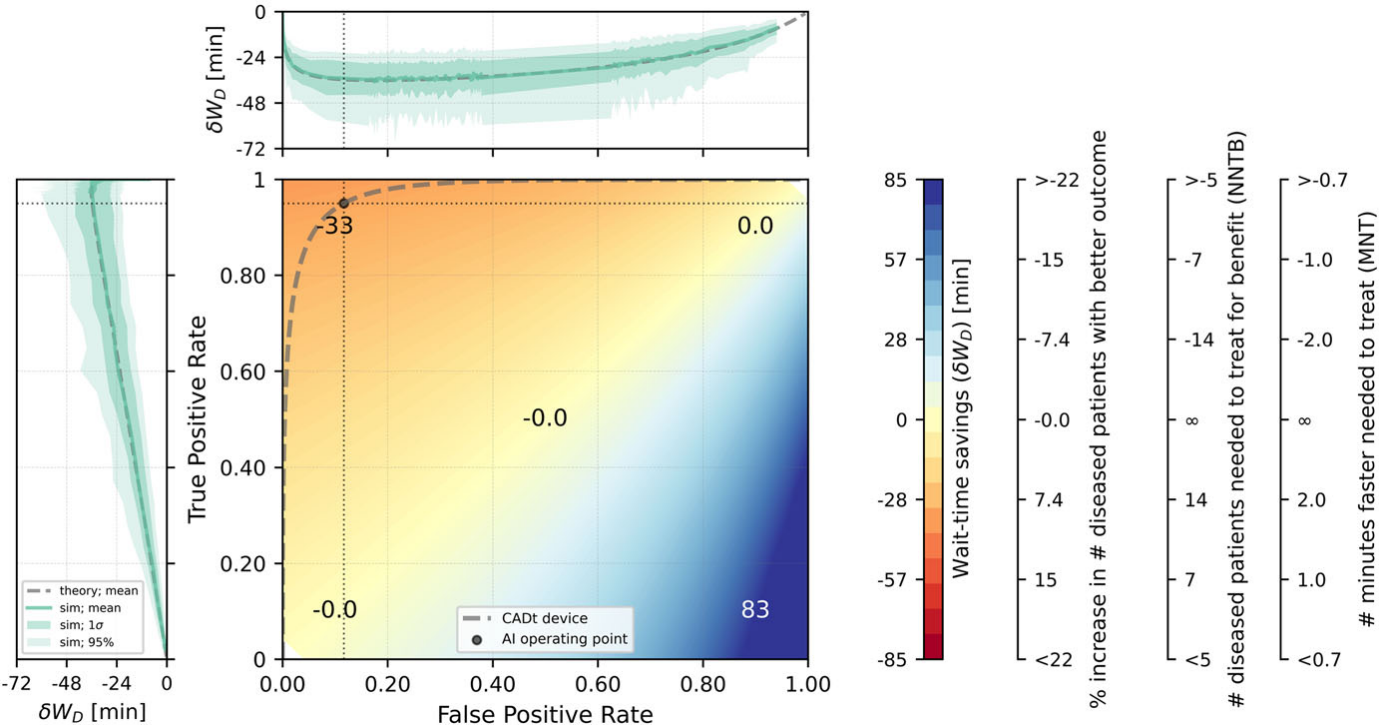
A summary ROC plot for evaluating both the diagnostic and wait-time-savings of a CADt device. The middle rainbow plot is an ROC space with an ROC curve (dashed dark gray) of a theoretical CADt device. Color map represents theoretical mean time savings ($\delta W_D$) per diseased patient image, assuming a disease prevalence of 10% in a relatively busy hospital (traffic intensity of 0.8) with only one radiologist and no interrupting patient images. The radiologist’s average reading times for diseased ($1/\mu _D$) and non-diseased ($1/\mu _{ND}$) patient images are both set at 10 min. Positive $\delta W_D$ (blue region) means an overall time delay for diseased patient images, and negative $\delta W_D$ (red region) means an overall time savings. The values printed on the color map are the $\delta W_D$’s at the corresponding points of false-positive and true-positive rates. The dot represents the pre-determined AI operating point ($\text {Se} = 95\%, \text {Sp}=89\%$). Top plot represents the theoretical $\delta W_D$ (dashed gray) along the ROC curve as a function of false-positive rate, and left plot represents the same theoretical $\delta W_D$ (dashed gray) along the curve but as a function of true-positive rate. The green solid line represents the mean time savings along the ROC curve obtained from simulation. The darker and lighter shaded areas indicate the 68% and 95% ranges from simulation around the mean time savings. The black dotted vertical and horizontal lines indicate that the theoretical mean time-saving for diseased patients is roughly 36 min at the given operating point. The color axis is translated to stroke patient outcome metrics based on Table 12 in Supplementary Content (Supplementary 2) of [12]

To summarize the diagnostic and time-saving ability of a CADt device, we propose a summary plot as shown in Fig. 10. This plot is built upon a traditional receiver operating characteristic (ROC) analysis [10], in which the ROC curve characterizes the diagnostic performance of the CADt device. For a given radiologist workflow defined by a set of parameters, every point of false-positive rate (FPR) and true-positive rate (TPR) in the ROC space has an expected mean time savings per diseased patient image, $\delta W_D$, which is presented by the color map. The diagnostic performance of a CADt device is near ideal in the top left corner of the ROC space, where $\delta W_D$ is the most negative.

To show the wait-time-savings of a CADt device, $\delta W_D$ along the ROC curve is plotted as a function of FPR (top) and TPR (left). At (FPR, TPR) = (0, 0), $\delta W_D$ is 0 min because all images are classified as AI-negative, i.e., no images are prioritized. As both FPR and TPR increase along the ROC curve, the amount of time savings $|\delta W_D|$ increases since most AI-positive cases are truly diseased patient images. As FPR and TPR continue to increase, the number of false-positive cases becomes dominant, reducing the device’s time-saving performance. When (FPR, TPR) = (1, 1), $\delta W_D$ goes back to 0 because all images are classified as AI-positive, and the system essentially has no priority classes.

For many time-sensitive conditions, patient outcomes are directly linked to how quickly a patient is seen and treated. Although our mathematical framework cannot calculate patient outcomes directly, the mean time-savings for diseased patient images $\delta W_D$ provides a quantitative metric of time savings, and thus, can be extrapolated to quantify aggregate patient outcome metrics in these scenarios. For example, if our disease of interest is large vessel occlusion (LVO) stroke, $\delta W_D$ color axis on the right side of Fig. 10 can be translated to three stroke patient outcome metrics. According to Table 12 in Supplementary Content (Supplementary 2) of [12], for every 15 min sooner that a patient is treated, 3.9% of stroke patients resulted in less disability. This can be translated to the two other common LVO stroke patient outcome metrics - the number of patients needed to treat for benefit (NNTB) and the number of minutes faster needed to treat (MNT). The relationships between $\delta W_D$ and LVO stroke patient outcome metrics are extrapolated linearly and shown in the three axes on the right side of Fig. 10. As a result, the optimal $\delta W_D$ along the ROC curve is roughly -40 min, which corresponds to approximately 11% increase in LVO stroke patients with less disability, more than 9 NNTB, and more than 1.4 MNT. Remember that these results depend on our assumed reading rates and traffic intensity. In the future we expect to gather clinical data to make more accurate estimates of reading rates, traffic intensity, and wait-time savings.

## Discussion

In this work, we apply queueing theory to quantify the average wait-time savings due to the deployment of a CADt device within a radiology clinic. The theoretical predictions agree with simulation results at 95% confidence intervals. We show that the wait-time-savings of a CADt device depends heavily on its diagnostic performance and the clinical settings. A CADt device with a typical AI diagnostic performance (95% sensitivity and 89% specificity) is most effective in a busy, short-staffed clinic. If a clinic is quiet with a low traffic intensity and/or has many radiologists to share the workload, images are read promptly by the radiologists and the addition of CADt does not further speed up the review of diseased patient images. In contrast, a CADt device is very effective if only one radiologist is available in a clinic with high traffic. This result is consistent with our clinical intuition, and our evaluation framework provides a quantification of how much time is saved for the diseased patient images.

When diseased patient images are the minority of the reading queue (i.e., disease prevalence less than 50%), a CADt device is more effective if a radiologist mean reading time for non-diseased images is longer than that for diseased images. This is because, when a non-diseased case takes the radiologist more time to complete (compared to a diseased case), a review of non-diseased case introduces a more noticeable delay (in comparison with a review of diseased case) to the rest of the diseased cases in the queue.

The roughly constant time-savings as a function of interrupting fraction *f* are also interesting. This behavior is likely because the amount of delay caused by interrupting patient images in a without-CADt scenario is similar to that in the with-CADt scenario. When taking the difference between the two scenarios, the effect to the mean wait-times is canceled out. This argument also holds for other state-dependent factors such as backlog—a radiologist is expected to work faster when the queue is long. Because its effect to the mean wait-time in both with- and without-CADt scenarios is the same, the impacts to mean time-savings is expected to be minimal. This highlights the fact that while the real clinical workflow is complicated, we may be able to simplify the queueing system when it comes to evaluating the time-savings (difference in mean wait-time) due to a CADt device.

Despite the agreement between theory and simulation, we acknowledge a few limitations in our workflow models.

*Number of radiologists in Model 2* One limitation is that Model 2 is restricted to one radiologist only. With more radiologists, the states in Figs. 6 and 7 would need to keep track of the disease status of every case that each of the $N_{\text {rad}}$ radiologists is reading. In addition, more truncated states may be needed to track which state the system must return to when the radiologists resume the review of the previously interrupted cases. We are investigating ways to simplify the information needed in a state by (e.g.) keeping track of the number of radiologists reading diseased cases and that reading non-diseased cases. Despite the challenges, we would like to point out that, given the same clinical settings and CADt diagnostic performance, having one radiologist reviewing images demonstrates the greatest potential time-saving benefits of a CADt device. Adding more radiologists to the system would only lower the wait-time-savings for diseased patient images.

*Exponential read-time assumption* In this work, radiologist reading time is assumed to be exponential, and we plan to assess the appropriateness of this assumption as we obtain real-world data from clinical sites. If it is found not to be exponential, it can be converted to a phase type distribution; for example, the EC approximation method can be used to approximate a non-exponential distribution to an Erlang–Coxian distribution up to the first three moments [11]. In addition, our mathematical approach did not take into account the difference in reading rates among true-positive (TP), true-negative (TN), false-positive (FP), and false-negative (FN) cases. For example, a radiologist may get confused when reviewing false-positive patients, taking longer to think if the device sees signs that the radiologist misses. In this case, $\mu _D$ and $\mu _{ND}$ would be further divided into four reading rates ($\mu _{TP}, \mu _{FP}, \mu _{TN}, \mu _{FN}$). It will further complicate the transition diagrams in Model 2, but the same concept is still applied. This consideration will be implemented in our future model expansion.

*Uncertainty based on simulation* It is also noted that we only applied queueing theory to compute the mean wait-time and relied on simulation for the confidence intervals around the mean. While methods such as distributional Little’s law [2, 9] can be applied to compute the uncertainty of waiting time per diseased patient, hypothesis testing typically depends on the uncertainty of the averaged device performance when evaluating a device. Therefore, to simplify the theoretical approach, we estimate the confidence intervals on mean wait-time differences by performing simulation trials.

*Alternative approaches* Our approach relies on the RDR approximation method to derive explicit formulae. As discussed in [6], the error rates of the RDR method increase as the number of priority classes and servers (radiologists) increases. While our workflow model considers at most three priority classes, the approximations from a large number of radiologists ($N_{\text {rad}}>$ 5) may not be accurate. Consequently, we are exploring alternative approaches that allow numerical evaluation of the stationary distribution of a multi-dimensional transition rate matrix. Although these numerical methods do not yield explicit formulae for wait-time performance metrics, they are more flexible for scaling the queueing system to accommodate more complex workflows.

*Workflows with multiple CADts and diseases* In this work, we only considered one CADt device triaging cases with one disease condition from all images in the reading queue; the AI-enabled CADt was trained to identify the one disease condition, and a patient image is either diseased or non-diseased with that disease condition. Under this consideration, CADt devices are evaluated based on the time-savings to the diseased subgroup, $\delta W_{\text {D}}$. However, if a radiologist queue consists of patient images with different conditions and/or if multiple CADts are used to triage patient cases with different conditions, the evaluation metric should also consider the potential time-savings for certain subgroups as well as the time-delay risks to other patient subgroups. Exploring multiple CADts on different conditions is our future work. One challenge of this model expansion is that most clinics have at most only one CADt device to triage the most time-sensitive condition in the radiologist queue, and we are unclear how a clinical site may orchestra the prioritization due to different CADt devices. For example, with two CADts, one site might put all AI-positive cases from both devices into the AI-positive priority class. Another site might triage one disease over another; AI-positive cases from device A has a higher priority than those from device B. Therefore, our next step is to have a better understanding of a potential workflow model a clinic may adopt with more than one CADt device. However, we would like to emphasize that estimating time savings based on a single disease CADt, is highly relevant, as most FDA-cleared devices include only prioritization for a single disease condition.

## Conclusion

We applied queueing theory, specifically the recursive dimensionality reduction method, to evaluate the time-saving performance of a computer-aided triage and notification (CADt) device. We investigated two workflow models from which the mean waiting time per patient image is theoretically calculated with and without a CADt device. The effect on the time-saving to diseased patient images due to various parameters (including disease prevalence, patient arrival rate, radiologist reading rate, number of radiologists on-site, AI sensitivity and specificity, as well as the presence of interrupting patient images) are studied. Agreement between theoretical and simulation results is reasonable for a wide range of parameter values. Our results show that a CADt device is more effective in triaging diseased patient images in a busy, short-staffed clinic. All software developed for theoretical calculations and simulations are publicly available on the Github [18].

## Figures and Tables

**Fig. 11:**
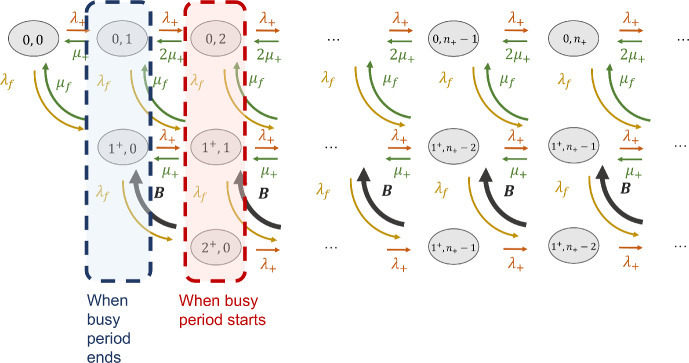
The transition diagram to calculate inter-level passage times within the AI-positive class (middle priority) in Model 1 in a with-CADt scenario

**Fig. 12:**
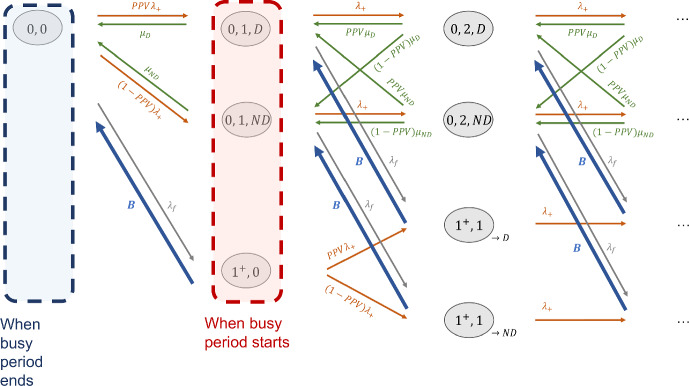
The transition diagram to calculate inter-level passage time within the AI-positive class in Model 2 in a with-CADt scenario

## Markov chain matrices

The appendix section provides the matrices involved for the two radiologist workflow models discussed in Sect. 2.2.

### Model 1 in without-CADt scenario

The transition rate matrix $M_{1_{\text {noCADt}}}$ is built upon Fig. 3.

16 $$\begin{aligned} M_{1_{\text {noCADt}}} = \left[ \begin{array}{c|c|c|c|c} B_{00} &  B_{01} &  &  &  \\ \hline B_{10} &  A_{1} &  A_{2} &  & \\ \hline &  A_0 &  A_{1} &  A_{2} &  \\ \hline &  &  A_{0} &  A_{1} &  \ddots \\ \hline &  &  &  \ddots &  \ddots \\ \end{array} \right] , \end{aligned}$$

where

$$\begin{aligned} A_0&= \begin{pmatrix} 2\mu _{\text {non}f} &  0 &  0 &  0\\ 0 &  \mu _{\text {non}f} &  0 &  0\\ 0 &  0 &  0 &  0\\ 0 &  0 &  0 &  0 \end{pmatrix}, \\ A_1&= \begin{pmatrix} * &  \lambda _f &  0 &  0\\ \mu _f &  * &  \lambda _f &  0\\ 0 &  t_1 &  * &  t_{12} \\ 0 &  t_2 &  0 &  * \end{pmatrix}, \\ A_2&= \begin{pmatrix} \lambda _{\text {non}f} &  0 &  0 &  0 \\ 0 &  \lambda _{\text {non}f} &  0 &  0 \\ 0 &  0 &  \lambda _{\text {non}f} &  0 \\ 0 &  0 &  0 &  \lambda _{\text {non}f} \end{pmatrix}, \end{aligned}$$

17

### Model 1 in with-CADt scenario

This scenario has six busy periods ($B_1$ to $B_6$). For each busy period, we first calculate its conditional probability and the first three moments of the inter-level passage times using Fig. 11. And the corresponding transition probability matrix $P_1$ is given below.

18 $$\begin{aligned} P_1 = \left[ \begin{array}{c|c|c|c|c} \mathcal {L}_{1} &  \mathcal {F}_{1} &  &  & \\ \hline \mathcal {B}_{2} &  \mathcal {L}_{2} &  \mathcal {F}_{2} &  & \\ \hline &  \mathcal {B}_{3} &  \mathcal {L}_{3} &  \mathcal {F}_{3} &  \\ \hline &  &  \mathcal {B}_{4} &  \mathcal {L}_{4} &  \ddots \\ \hline &  &  &  \ddots &  \ddots \\ \end{array} \right] , \end{aligned}$$

where

19

With $P_1$, the conditional probabilities and first three moments of inter-level passage times for all six busy periods are computed according to Appendix A of [6]. For most busy periods, a two-phase Coxian distribution is sufficient for the Erlang–Coxian (EC) approximation, resulting in three *t*-parameters. However, the approximation of $B_2$ and $B_5$ requires one Erlang phase, and two additional parameters $t_0$ and $t_{01}$ are required.

Let $t^{(i)}_j$ denote the $t_j$-parameter for a busy period $B_i$. The transition rate matrix $M_{1_{\text {CADt}}}$ for the AI-negative class from Fig. 5 is given by

20 $$\begin{aligned} M_{1_{\text {CADt}}} = \left[ \begin{array}{c|c|c|c} B_{00} &  B_{01} &  &  \\ \hline B_{10} &  A_{1} &  A_{2} &  \\ \hline &  A_0 &  A_{1} &  \ddots \\ \hline &  &  A_{0} &  \ddots \\ \hline &  &  &  \ddots \\ \end{array} \right] . \end{aligned}$$

All *A* sub-matrices are $17\times 17$. Here, $\mathbb {0}_{14}$ denotes a $14\times 14$ zero matrix, and $\mathbb {I}_{17}$ is a $17\times 17$ identity matrix.

21

where for $$i = 1, 3, 4, 6$$,

$$\begin{aligned} \mathbf {p_i} = \begin{pmatrix} p_i&0 \end{pmatrix}, \hspace{5.0pt}\textbf{t}^{(i)} = \begin{pmatrix} t_1^{(i)} \\ t_2^{(i)} \end{pmatrix}, \hspace{5.0pt}\mathbb {T}^{(i)}_k = \begin{pmatrix} * &  t_{12}^{(i)} \\ &  * \end{pmatrix}. \end{aligned}$$

For $i = 2, 5$, because of the extra Erlang phase, the sub-matrices have an extra row and/or column.

$$\begin{aligned} \mathbf {p_i} = \begin{pmatrix} p_i&0&0 \end{pmatrix}, \hspace{5.0pt}\textbf{t}^{(i)} = \begin{pmatrix} t_0^{(i)} \\ t_1^{(i)} \\ t_2^{(i)} \end{pmatrix}, \hspace{5.0pt}\mathbb {T}^{(i)}_k = \begin{pmatrix} * &  t_{01}^{(i)} &  \\ &  * &  t_{12}^{(i)} \\ &  &  * \end{pmatrix}. \end{aligned}$$

Similarly, the boundary *B* sub-matrices are given below.

22

### Model 2 in without-CADt scenario

The transition rate matrix $M_{2_{\text {noCADt}}}$ for Fig. 6 is given below.

23 $$\begin{aligned} M_{2_{\text {noCADt}}} = \left[ \begin{array}{c|c|c|c|c} B_{00} &  B_{01} &  &  &  \\ \hline B_{10} &  A_{1} &  A_{2} &  & \\ \hline &  A_0 &  A_{1} &  A_{2} & \\ \hline &  &  A_{0} &  A_{1} &  \ddots \\ \hline &  &  &  \ddots &  \ddots \\ \end{array} \right] , \end{aligned}$$

where

24

where $\lambda _{\text {non}f}$ refers to the arrival rate of non-interrupting subgroup.

### Model 2 in with-CADt scenario

This scenario has three busy periods ($B_1$ to $B_3$). For each busy period, we calculate its conditional probability and the first three moments of the inter-level passage times using Fig. 12 and the corresponding transition probability matrix $P_2$.

25 $$\begin{aligned} P_2 = \left[ \begin{array}{c|c|c|c|c} \mathcal {L}_{1} &  \mathcal {F}_{1} &  &  & \\ \hline \mathcal {B}_{2} &  \mathcal {L}_{2} &  \mathcal {F}_{2} &  & \\ \hline &  \mathcal {B}_{3} &  \mathcal {L}_{3} &  \mathcal {F}_{3} &  \\ \hline &  &  \mathcal {B}_{4} &  \mathcal {L}_{4} &  \ddots \\ \hline &  &  &  \ddots &  \ddots \\ \end{array} \right] , \end{aligned}$$

where

26

Each of the three busy periods has a set of *t*-parameters (Eq. 10) approximated from a two-phase Coxian distribution.

Let $t^{(i)}_j$ denote the $t_j$ parameter for the busy period $B_i$. The transition rate matrix $M_{2_{\text {CADt}}}$ for the AI-negative subgroup (Fig. 7) is given by

27 $$\begin{aligned} M_{2_{\text {CADt}}} = \left[ \begin{array}{c|c|c|c} B_{00} &  B_{01} &  &  \\ \hline B_{10} &  A_{1} &  A_{2} &  \\ \hline &  A_0 &  A_{1} &  \ddots \\ \hline &  &  A_{0} &  \ddots \\ \hline &  &  &  \ddots \\ \end{array} \right] \end{aligned}$$

The $14\times 14$ *A* sub-matrices are defined below, where $\mathbb {0}_{12}$ denotes a $12\times 12$ zero matrix, and $\mathbb {I}_{14}$ is a $14\times 14$ identity matrix.

28

where for a busy period ${\textbf {B}}_i$,

29 $$\begin{aligned} \textbf{p} = \begin{pmatrix} 1&0 \end{pmatrix}, \hspace{5.0pt}\textbf{t}^{(i)} = \begin{pmatrix} t_1^{(i)} \\ t_2^{(i)} \end{pmatrix}, \hspace{5.0pt}\mathbb {T}^{(i)}_k = \begin{pmatrix} * &  t_{12}^{(i)} \\ 0 &  * \end{pmatrix}. \end{aligned}$$

The boundary *B* matrices, on the other hand, are

30

where

31
